# Unified In‐Memory Hardware Security Enabled by an Entropy Landscape in a Memristor Crossbar

**DOI:** 10.1002/smll.75550

**Published:** 2026-09-02

**Authors:** Jinwoo Park, Hyungjin Kim

**Affiliations:** ^1^ Division of Materials Science and Engineering and Department of Semiconductor Engineering Hanyang University Seoul South Korea

**Keywords:** encryption and decryption, hardware security, memristor crossbar array, physical unclonable function (PUF), privacy‐preserving mutual authentication (PPMA), true random number generator (TRNG)

## Abstract

Memristor‐based hardware security primitives have attracted attention owing to their intrinsic variability and stochastic switching dynamics; however, most prior demonstrations implement physical unclonable functions (PUFs), true random number generators (TRNGs), and cryptographic operations as functionally separated modules. Here, we present a unified hardware security platform in which PUF, TRNG, and in‐memory encryption are co‐realized within a single memristor crossbar array. Post‐fabrication annealing reshapes the high‐resistance‐state landscape into a stable dual‐entropy material state, simultaneously enabling static device‐to‐device variability for PUF operation and dynamic random telegraph noise for TRNG generation. Leveraging this entropy‐engineered landscape, vector–matrix multiplication‐based encryption is directly implemented within the array and combined with lookup‐table‐assisted decryption, allowing encryption to operate as an intrinsic in‐memory physical process. The integrated system demonstrates collision‐resilient encryption, robust decryption under device variations, high‐quality randomness, and reliable privacy‐preserving mutual authentication. This work establishes a materials‐driven approach to compact, low‐power, and intrinsically secure in‐memory hardware architectures for next‐generation Internet‐of‐Things applications.

## Graphical abstract

Post‐fabrication annealing tailors the defect landscape of a memristor crossbar to provide complementary static and dynamic entropy within a single physical platform. The resulting device‐to‐device variability and random telegraph noise enable PUF and TRNG functionalities, while analog in‐memory computation further supports hardware‐embedded cryptographic processing, establishing a compact materials‐to‐security framework for secure edge systems.

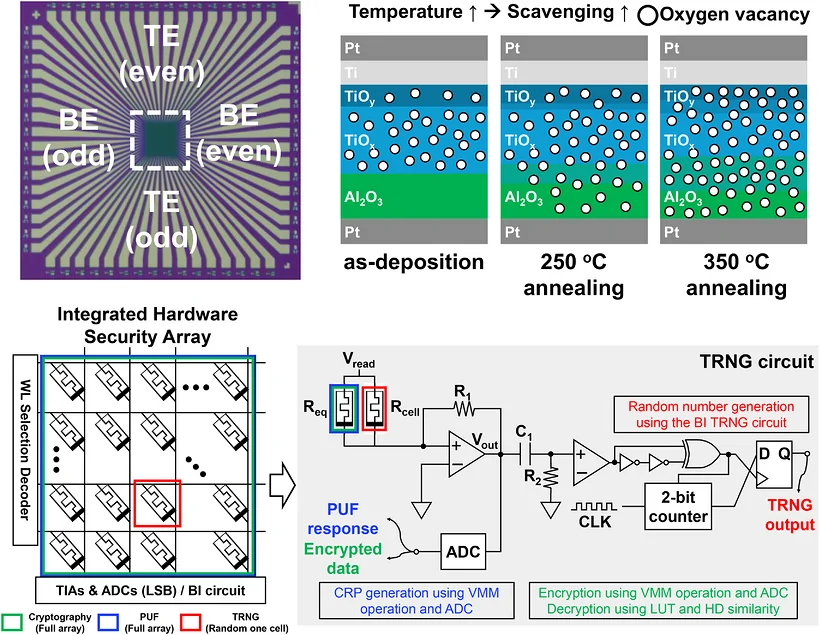

## 1 Introduction

The rapid proliferation of Internet‐of‐Things (IoT) devices has intensified the demand for lightweight, low‐power, and highly secure hardware platforms capable of protecting sensitive data exchanged over distributed and often untrusted networks [1, 2, 3]. Conventional cryptographic systems rely on digitally stored secret keys and algorithmic encryption executed by peripheral logic, which exposes systems to key extraction, side‐channel attacks, and machine‐learning‐assisted modeling attacks [4, 5, 6]. These vulnerabilities become particularly critical in resource‐constrained IoT environments, where stringent power, area, and cost limitations restrict the deployment of complex security circuitry. As a result, there has been growing interest in hardware‐embedded security primitives that derive their security directly from intrinsic physical properties rather than from stored digital secrets.

Physical unclonable functions (PUFs) and true random number generators (TRNGs) represent two fundamental building blocks for hardware security. PUFs exploit intrinsic device‐to‐device variations to generate unique and unclonable identities, while TRNGs rely on stochastic physical processes to produce unpredictable random numbers required for cryptographic protocols [7, 8, 9, 10, 11, 12, 13, 14, 15, 16, 17, 18]. In‐memory computing architectures have been realized using a wide range of memory technologies, including flash, phase‐change memory, ferroelectric devices, and resistive switching memories, aiming to exploit intrinsic physical properties for computation and security tasks [19, 20, 21, 22, 23, 24, 25, 26, 27, 28, 29, 30]. Among these candidates, memristors have attracted considerable attention for in‐memory computing applications, including hardware security due to their nanoscale footprint, high scalability, and rich physical non‐idealities, including conductance variability, stochastic filament formation, and random telegraph noise (RTN) [31, 32, 33, 34, 35, 36, 37, 38, 39, 40, 41]. These characteristics have enabled a wide range of memristor‐based PUFs and TRNGs, demonstrating promising entropy quality and robustness at both the device and array levels. Beyond entropy generation, memristor crossbar arrays can offer an additional and highly attractive capability: massively parallel vector–matrix multiplication (VMM) based on analog current superposition. This property has been extensively explored for in‐memory computing and neuromorphic applications [42], enabling energy‐efficient parallel computation directly within the memory fabric. Beyond computational functionality, incorporating hardware security primitives into memristor‐based computing platforms could further extend their functionality toward secure and multifunctional memory‐centric systems. More recently, this analog computing capability has been extended to hardware security, enabling in‐memory encryption schemes that leverage physical current superposition rather than conventional digital logic. Such approaches provide an opportunity to realize cryptographic operations as intrinsic physical processes, potentially reducing hardware overhead and minimizing the exposure of sensitive intermediate data.

Despite these advances, most prior memristor‐based security demonstrations have implemented PUF, TRNG, and cryptographic functionalities as separate hardware primitives [43, 44, 45]. More recently, significant progress has been made toward integrating multiple security functions, including unified PUF and TRNG operation within memristive arrays [46, 47, 48, 49, 50]. In parallel, other studies have explored in‐memory cryptographic operations using memristive devices, demonstrating the potential of memory arrays for hardware‐level data protection [51, 52, 53, 54, 55, 56]. These developments have established complementary approaches toward entropy generation and in‐memory cryptographic processing, while their integration within a common memory platform remains relatively less explored. In addition, the relationship between materials‐level entropy characteristics and array‐level security functionality remains relatively underexplored. Building upon these advances, an integrated materials‐to‐architecture approach that combines device‐derived entropy generation with analog in‐memory cryptographic processing offers an opportunity to further consolidate multiple security functions within a common crossbar platform.

A unified security primitive capable of generating challenge–response pairs (CRPs), producing high‐quality randomness, and performing in‐memory encryption and decryption within a single memristor crossbar array would therefore offer a compelling solution to these challenges. In such an architecture, intrinsic device‐to‐device variability can be exploited to generate strong PUF responses, while dynamic noise sources, such as RTN, provide high‐entropy randomness without requiring dedicated random‐number hardware. At the same time, the analog VMM capability of crossbar arrays enables lightweight and energy‐efficient encryption and decryption directly within the memory fabric, eliminating the need to expose sensitive intermediate data to peripheral logic [57]. By physically co‐localizing these security primitives within a single array, a fully hardware‐embedded, privacy‐preserving mutual authentication (PPMA) protocol can be realized, in which device identity, random challenge generation, and data protection are all achieved without storing static secret keys or relying on vulnerable digital logic [58, 59, 60, 61]. Such a unified in‐memory security architecture reduces hardware overhead while enhancing system‐level robustness, making it particularly well suited for lightweight IoT deployments.

In this work, we present an integrated memristor crossbar array–based hardware security platform that co‐realizes PUF, TRNG, and analog in‐memory encryption within a single oxide memristor crossbar array. Distinct from prior approaches that treat these security primitives as functionally independent modules, we show that post‐fabrication annealing fundamentally reshapes the high‐resistance‐state (HRS) landscape of the memristor array into a stable dual‐entropy material state, simultaneously supporting static device‐to‐device variability for PUF operation and dynamic RTN for TRNG generation within the same physical stack. Through systematic annealing temperature optimization, a forming‐free switching regime with a 100% array yield and a well‐stabilized HRS distribution is achieved, providing a robust materials foundation for entropy generation. Utilizing this materials‐engineered entropy platform, we further demonstrate VMM‐based in‐memory encryption together with look‐up table (LUT)/Hamming distance (HD)‐assisted decryption, reducing reliance on externally stored digital secret keys. Finally, by integrating the unified PUF, TRNG, and cryptographic primitives, a PPMA protocol is experimentally validated at the array level, highlighting the potential of the proposed architecture as a compact, low‐power, and scalable hardware security solution for lightweight IoT applications.

## 2 Results and Discussion

### 2.1 Device Fabrication and Array‐Level Switching Characteristics

The process flow used to fabricate the 32 × 32 memristor crossbar array is shown in Figure 1a. After patterning the bottom electrodes (BEs) on a 4‐inch wafer with a 300‐nm thermally grown SiO_2_ layer, the Al_2_O_3_ main switching layer and the TiO_x_/TiO_y_ multilayer stack were subsequently deposited. Following this, the top electrodes (TEs) were patterned in a direction orthogonal to the BEs, ensuring adequate isolation between adjacent cells. As the final process step, the devices underwent post‐fabrication annealing under an N_2_ atmosphere for 10 min to thermally activate the switching layer and modulate the HRS landscape, and their electrical characteristics were systematically evaluated across different annealing temperatures. Figure 1b shows a top‐view optical microscopy (OM) image of the fabricated 32 × 32 memristor crossbar array, an enlarged scanning electron microscopy (SEM) view of a representative cross‐point cell, and a transmission electron microscopy (TEM) image confirming the integrity of the stacked layer structure. The fabricated array was physically connected to a custom hardware measurement setup for array‐level electrical characterization. Figure 1c presents a schematic of the hardware interconnection configuration operated through command instructions issued by a control PC. The 64 electrode pads of the array were contacted using a 64‐pin probe card, and voltage signals from a Keysight B1500A semiconductor parameter analyzer were applied through measurement paths configured by a Keysight B34980A switching matrix.

**FIGURE 1:**
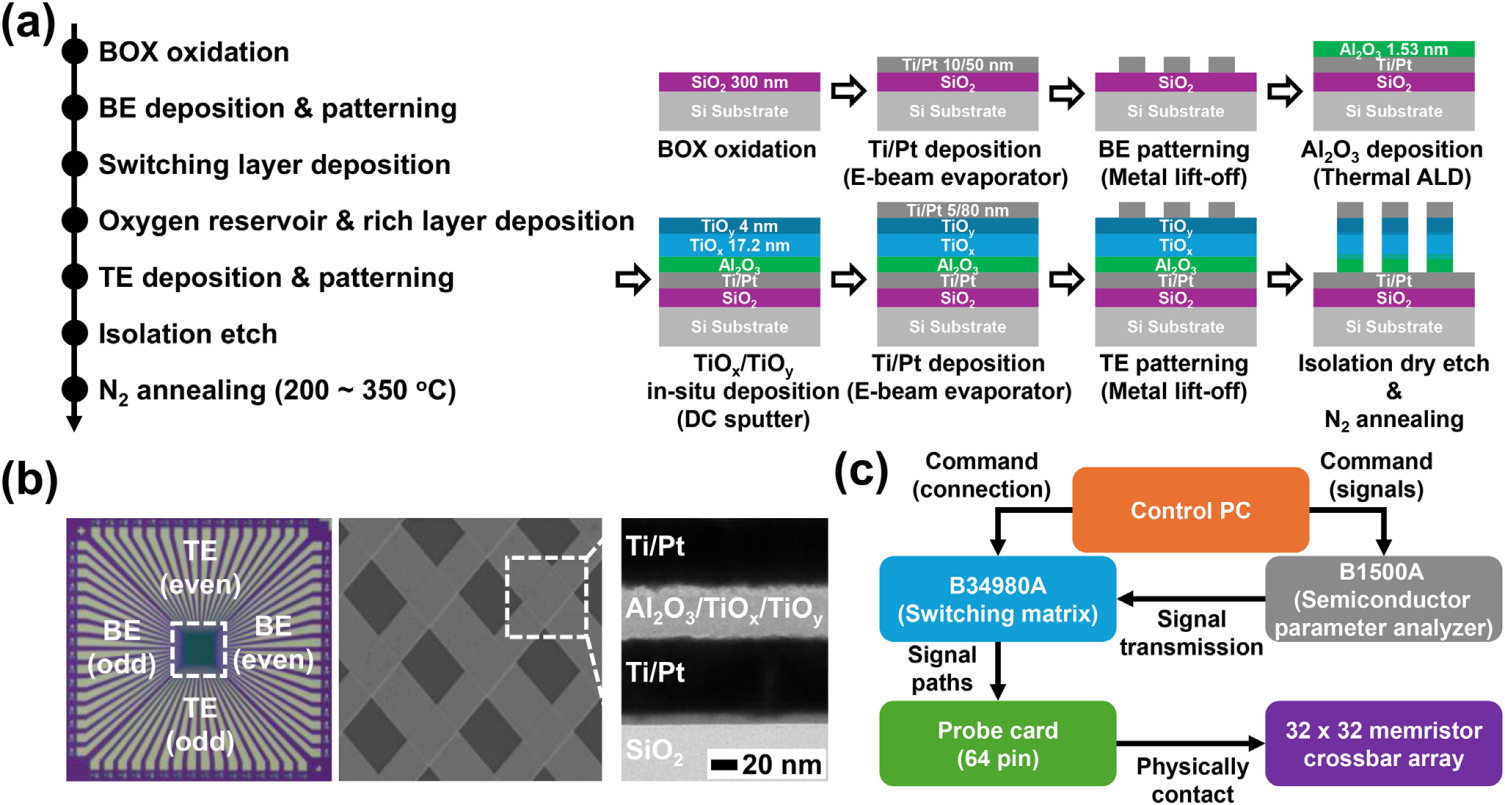
Crossbar array fabrication and measurement. (a) Fabrication process flow of the 32 × 32 memristor crossbar array, including BE patterning, deposition of the Al_2_O_3_ switching layer and TiO_x_/TiO_y_ multilayer stack, TE formation, and post‐fabrication annealing under an N_2_ atmosphere to engineer the resistance landscape. (b) OM image of the fabricated array, enlarged SEM image of a representative cross‐point cell, and cross‐sectional TEM image showing the stacked structure and layer uniformity. (c) Schematic of the hardware measurement configuration, where the array is contacted via a 64‐pin probe card and characterized using a semiconductor parameter analyzer and switching matrix controlled by a host PC, enabling array‐level VMM‐based security evaluation.

To investigate the impact of post‐fabrication annealing on device behavior and array yield, the electrical characteristics obtained at each annealing temperature were systematically analyzed. Figure 2a illustrates a schematic comparison between the as‐deposited array and its thermally activated state after annealing. While the as‐deposited array exhibits a relatively uniform pristine resistance distribution, annealing induces a broader resistance distribution due to temperature‐driven thermal activation. In the as‐deposited condition, the absence of conductive paths makes the array prone to hard breakdown during repeated switching cycles due to stochastic overshoot currents during forming, driven by oxygen drift under high electric fields in the pristine switching layer. In contrast, post‐fabrication annealing can thermally activate the switching layer through interfacial scavenging and vacancy generation, promoting stable soft breakdown and preserving reliable switching behavior over multiple cycles. Figure 2b shows the initial resistance distributions of the as‐deposited 32 × 32 memristor crossbar array and those annealed at temperatures ranging from 200°C to 350°C. As the annealing temperature increases, the initial resistance progressively decreases, yielding average resistance values of 432.2 kΩ (σ = 27.42 kΩ), 335.84 kΩ (σ = 38.65 kΩ), 196.29 kΩ (σ = 57.45 kΩ), 114.08 kΩ (σ = 23.89 kΩ), and 24.21 kΩ (σ = 10.94 kΩ) for the as‐deposited, 200°C, 250°C, 300°C, and 350°C conditions, respectively. Figure 2c summarizes the proportion of thermally activated cells at each annealing temperature, defined using a reference resistance of 250 kΩ at a read voltage (*V*_read_) of 0.2 V, which is higher than the average HRS resistance of the as‐deposited array. Based on this activation criterion, annealing temperatures of 250°C and above result in substantial thermal activation, enabling forming‐free switching behavior across most cells. Consistent with this observation, the distribution of the first set voltage, including the forming process, becomes progressively narrower with increasing annealing temperature, as shown in Figure 2d. The average first set voltages for the 32 × 32 arrays are 1.81 V (σ = 0.31 V), 1.71 V (σ = 0.24 V), 1.48 V (σ = 0.28 V), 1.47 V (σ = 0.24 V), and 1.46 V (σ = 0.21 V) for the as‐deposited, 200°C, 250°C, 300°C, and 350°C conditions, respectively. The average set voltage decreases markedly up to the 250°C annealing condition, while only marginal additional reductions are observed at higher annealing temperatures, suggesting that the thermal activation effect approaches saturation beyond 250°C.

**FIGURE 2:**
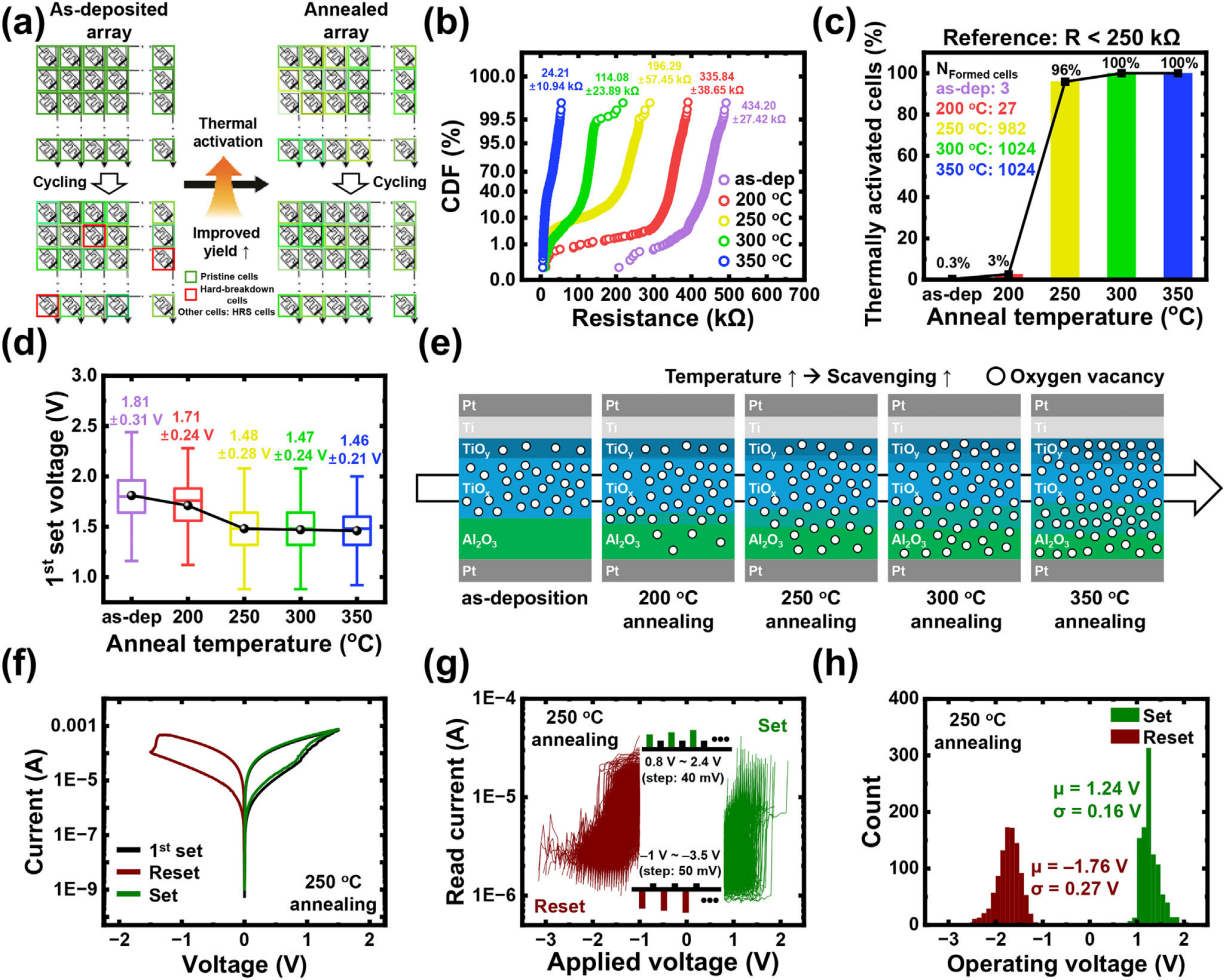
Annealing‐induced thermal activation of memristor crossbar and switching stabilization. (a) Schematic comparison of as‐deposited and annealed device states, illustrating thermally activated vacancy formation and interfacial reactions. (b) Initial resistance distributions at different annealing temperatures, showing progressive thermal activation and reduced high‐resistance‐state variability. (c) Fraction of thermally activated cells vs. annealing temperature, indicating transition toward forming‐free operation above 250°C. (d) First set‐voltage distributions including forming behavior, demonstrating narrowing of voltage spread with optimized annealing. (e) Illustration of interfacial scavenging and oxygen vacancy generation responsible for stabilized switching dynamics. (f) Representative *I*–*V* characteristics of the 250°C‐annealed device exhibiting forming‐free behavior and symmetric switching. (g) Switching characteristics under ISPP with half‐*V* scheme, confirming controlled programming behavior across cycles. (h) Set and reset voltage distributions across 1,024 cells, validating uniform array‐level switching essential for reliable entropy generation.

Figure 2e illustrates how higher annealing temperatures initiate interfacial reactions that thermally activate the devices and induce interlayer scavenging. Elevated annealing temperatures promote thermally driven interfacial overlap between adjacent layers, leading to enhanced oxygen diffusion and the generation of additional oxygen vacancies, which increase the susceptibility of the devices to forming. It is important to note that the post‐fabrication annealing in this study was conducted at relatively low temperatures (200°C–350°C), below the thermal budgets generally associated with pronounced crystallization of ALD Al_2_O_3_ and TiO_x_‐based films [62, 63]. Consequently, the annealing‐induced device stabilization observed in this temperature range is primarily attributed to interfacial oxygen scavenging and the associated modification of oxygen‐vacancy‐related defect states, rather than to macroscopic crystallization of the functional layers [64]. Therefore, the fraction of cells exhibiting switching behavior during the first reset operation increases with annealing temperature, consistent with an increased population of thermally formed devices (Figure S1). However, insufficient annealing can still allow stochastic current overshoot during the forming process, increasing the risk of hard breakdown. In contrast, excessive annealing may induce overly strong interfacial reactions not only at the Al_2_O_3_/TiO_x_ interface but also at the TiO_x_/TiO_y_ interface, leading to excessive thermal forming and a higher probability of reset failure. These competing effects are further supported by the array‐level resistance distributions shown in Figure S2. To quantitatively evaluate array‐level switching robustness, the switching capability of every memristor cell was assessed by comparing the initial resistance distribution with that obtained after 10 switching cycles, as shown in Figure S3. Across all annealing conditions, high array yields exceeding 99% were achieved, indicating that post‐fabrication annealing effectively suppresses catastrophic switching failures at the array level. Notably, the highest yield of 100% was obtained at the 250°C annealing condition, while both lower and higher annealing temperatures exhibited a small fraction of failed cells, reflecting the competing failure mechanisms associated with insufficient and excessive thermal activation. As illustrated in Figure S4, insufficient annealing predominantly led to hard breakdown, whereas excessive annealing resulted in reset failures or cells remaining stuck in the low‐resistance state (LRS). These distinct failure modes further confirm the existence of an optimal annealing window, within which thermal activation is sufficient to stabilize switching behavior without inducing excessive interfacial reactions.

Figure 2f shows the current–voltage (*I*–*V*) characteristics of memristors in the array annealed at 250°C, while the corresponding characteristics under other annealing conditions are provided in Figure S5. Owing to thermal activation, both the initial and subsequent set operations exhibit nearly identical forming‐free switching behavior, with operating voltages confined within ±1.5 V. Physically, the resistive switching behavior can be understood in terms of electric‐field‐driven oxygen‐ion redistribution and the associated modulation of oxygen vacancies within the Al_2_O_3_ switching layer. To substantiate the material basis for the oxygen‐mediated switching behavior, X‐ray photoelectron spectroscopy (XPS) analysis was performed to evaluate the initial chemical states of the constituent TiO_x_ and TiO_y_ layers in their as‐deposited conditions (Figure S6). The XPS analysis yields O/Ti ratios corresponding to approximately TiO_1.67_ and TiO_1.75_ for the TiO_x_ and TiO_y_ layers, respectively. The lower O/Ti ratio of the TiO_x_ layer indicates a greater degree of oxygen deficiency and a higher relative abundance of oxygen‐vacancy‐related defects compared with the TiO_y_ layer. This compositional difference supports the role of the oxygen‐deficient TiO_x_ layer as an oxygen reservoir, facilitating oxygen‐ion redistribution during resistive switching. Based on the proposed mechanism, when a positive set voltage is applied to the top electrode, oxygen ions migrate from the Al_2_O_3_ switching layer toward the oxygen‐deficient TiO_x_ layer, which serves as an oxygen reservoir. The resulting increase in oxygen‐vacancy concentration within the Al_2_O_3_ layer promotes the formation of a conductive filament, switching the device to the LRS. Conversely, under the negative reset bias, oxygen ions migrate back toward the Al_2_O_3_ layer and recombine with oxygen vacancies, disrupting the conductive filament and returning the device to the HRS. To further examine the filamentary nature of this switching mechanism, the conduction characteristics of the HRS and LRS were analyzed using *I*–*V* fitting. As shown in Figure S7a, the HRS is well described by Schottky emission, indicating interface‐limited transport after disruption of the conductive pathway. In contrast, the LRS exhibits approximately Ohmic behavior with a slope close to 1 (Figure S7b), consistent with conduction through a filamentary pathway. The same overall transport behavior is observed across the investigated annealing conditions, supporting a common filamentary switching mechanism.

In a small number of cells, local variations in the annealing condition led to partial thermal forming, as shown in Figure S8a, resulting in devices that initiated switching from the first reset operation rather than requiring an initial set transition. The set and reset operations with incremental step pulse programming (ISPP) and the half‐*V* scheme for all cells in the 250°C‐annealed array are shown in Figure 2g. The set voltage pulses were applied from 0.8 to 2.4 V in 40 mV steps, while the reset pulses were swept from −1.0 to −3.5 V in 50 mV steps, with a fixed pulse width of 100 µs. The threshold currents for the set and reset operations were set to 8 µA and 2 µA, respectively. Based on these programming conditions, Figure 2h summarizes the operating voltage distributions for 1,024 memristor cells. The average set and reset voltages were 1.24 V (σ = 0.16 V) and –1.76 V (σ = 0.27 V), respectively, indicating stable and uniform switching behavior across the array. The resulting HRS and LRS distributions are shown in Figure S8b, while the corresponding endurance and retention characteristics are provided in Figure S8c,d. Importantly, while multilayer engineering is conventionally employed to confine conductive filament formation and improve switching uniformity, such spatial confinement does not eliminate the microscopic stochasticity associated with vacancy migration and filament rupture. In our device, the Al_2_O_3_ film serves as the primary filamentary switching layer, whereas the TiO_x_ and TiO_y_ layers facilitate stable switching by acting as an oxygen reservoir and an overshoot‐current suppressor, respectively. Because the TiO_x_/TiO_y_ multilayer exhibits substantially lower resistance than the Al_2_O_3_ layer, the dominant filamentary switching region is localized within the Al_2_O_3_ layer, as supported by c‐AFM analysis [65]. Stochastic oxygen‐vacancy redistribution during filament rupture leaves spatially nonuniform residual conductive paths in the HRS, giving rise to the static device‐to‐device conductance variation exploited for PUF operation. In contrast, stochastic electron capture and emission at localized vacancy‐related trap states near these residual conductive paths produces the dynamic RTN utilized for TRNG generation.

### 2.2 Collision‐Resilient In‐Memory Encryption Architecture

To demonstrate a lightweight hardware security architecture, we developed a 32 × 32 memristor crossbar array that integrates PUF, TRNG, and in‐memory cryptographic processing within a single hardware platform. Figure 3a illustrates the schematic of the integrated hardware security array based on the memristor crossbar architecture. The full array supports both cryptographic processing and PUF operations, while additionally enabling TRNG functionality by leveraging the RTN signal from a randomly selected memristor cell within the array. For encryption and decryption, the original 32 × 32 array was reconfigured into an 8 × *N* structure. In this architecture, increasing the column‐to‐row ratio statistically reduces encryption/decryption collisions by expanding the output dimensionality relative to the input space. Accordingly, the array was rearranged into an 8 × 128 configuration, where 8‐bit inputs were assigned to enable direct experimental verification of collision behavior and decryption accuracy at the array level. The 8‐bit input width represents a minimal experimental demonstrator rather than an intrinsic architectural limitation. Under this configuration, the voltage application sequence for each 8‐bit input is determined by the wordline (WL) selection decoder, and the subsequent VMM operation generates *N* bitline (BL) currents. These BL currents are converted into voltages through a transimpedance amplifier (TIA) and subsequently digitized by an analog‐to‐digital converter (ADC). Based on the ADC resolution, the final output bit is extracted as the least significant bit (LSB). The encrypted outputs are stored in an LUT, and the original input is recovered during decryption by identifying the LUT entry with the minimum HD. In the TRNG mode, the RTN signal from a randomly selected memristor cell serves as a dynamic entropy source. This signal is fed into the bias‐independent (BI) TRNG circuit, enabling the generation of high‐quality random numbers [66]. Unlike conventional digital encryption schemes, the proposed approach exploits analog current superposition and material‐level conductance dispersion inherent to the memristor crossbar array, thereby realizing encryption as an intrinsic in‐memory physical process rather than an algorithmic overlay.

**FIGURE 3:**
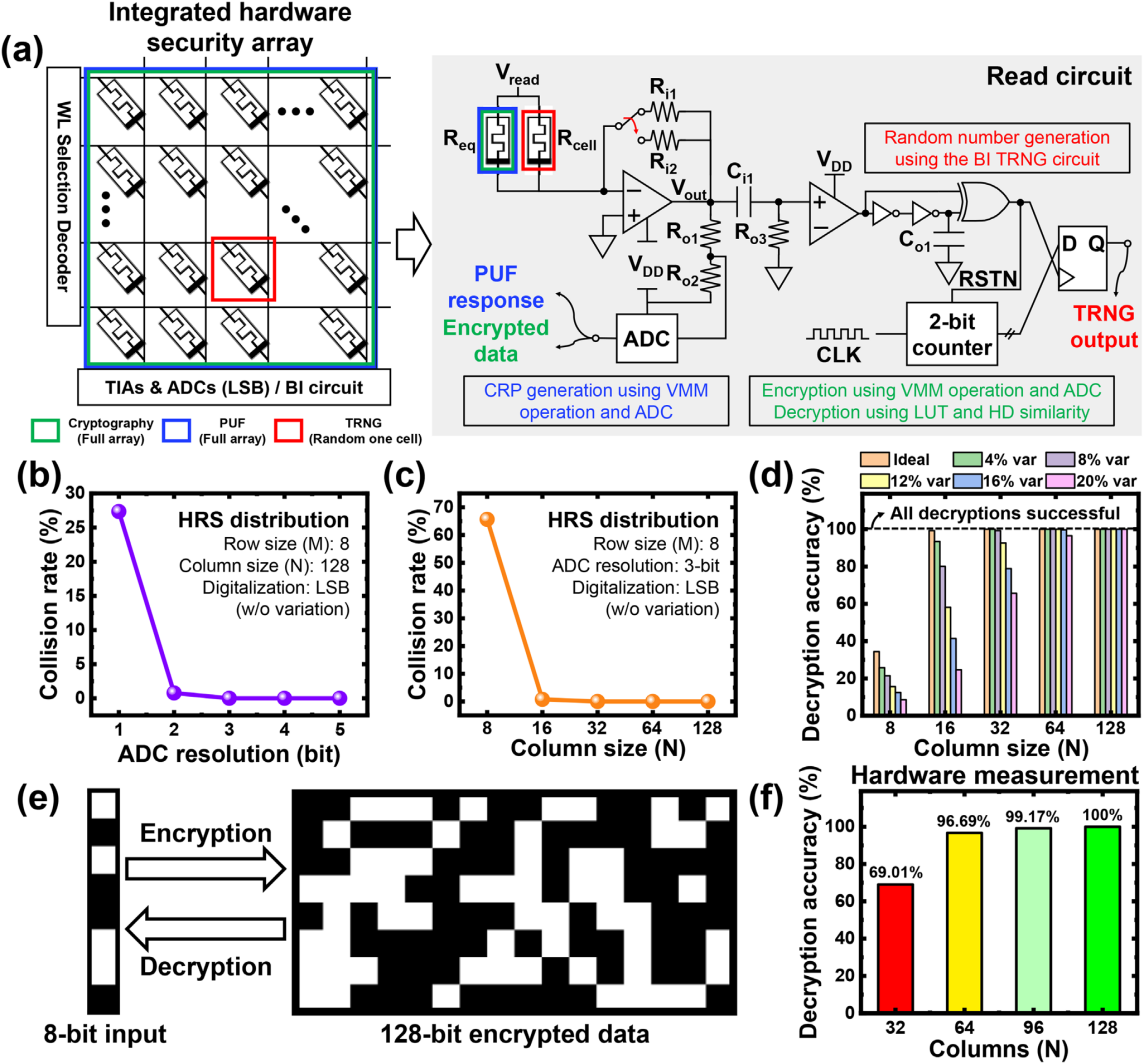
Collision‐resilient in‐memory encryption architecture. (a) Schematic of the unified crossbar architecture integrating PUF, TRNG, and encryption/decryption within a single array platform. (b) Collision rate of encrypted outputs vs. ADC resolution under the 8 × 128 configuration, showing that insufficient digitization precision leads to output ambiguity. (c) Collision rate as a function of column dimension *N*, demonstrating dramatic suppression of identical encrypted outputs as the effective physical bit space expands. (d) Decryption accuracy vs. *N* under varying conductance variation levels, highlighting enhanced robustness in larger column configurations. (e) Example of 8‐bit input mapped to a 128‐bit encrypted output and successful hardware decryption via HD matching. (f) Experimentally measured decryption accuracy across different column sizes, confirming that column scaling mitigates variation‐induced decryption errors.

To evaluate potential collisions among the encrypted data, we analyzed the consistency of the VMM‐based encryption outputs generated from the 8 × *N* crossbar array. As a first step, we examined the collision rate assuming the maximum reconstructed array dimension of 8 × 128 (1,024 cells), as shown in Figure 3b, and investigated its dependence on ADC resolution. At low ADC resolutions, insufficient representation of BL current information resulted in a substantial number of output collisions. In the extreme case of a 1‐bit ADC, the collision rate reached 27.34%, whereas no collisions were observed for any input pattern when the ADC resolution was 3 bits or higher. Figure 3c further shows the collision rate as a function of the column size *N* under a 3‐bit ADC assumption. For the square 8 × 8 configuration, the collision rate reached 65.63%, indicating that distinct input patterns were frequently mapped to identical outputs. As *N* increases and the effective output bit length expands, the collision probability decreases dramatically. In the ideal VMM operation, no collisions were observed once *N* reached 32, confirming that enlarging the column dimension effectively suppresses encryption ambiguity. This trend reflects the expansion of the effective physical bit space, which statistically lowers the probability of generating identical encrypted outputs.

However, the absence of collisions under ideal conditions does not necessarily guarantee flawless decryption in practical hardware, as conductance variations during VMM operations may cause deviations in the encrypted outputs. Figure 3d presents the decryption accuracies as a function of *N* under various assumed conductance variations, including both read‐related fluctuations and minor unintended state shifts. When *N* was 8, the high intrinsic collision rate resulted in poor decryption accuracy regardless of variation level. Although *N* = 32 achieved 100% accuracy under ideal conditions, an 8% variation already led to decryption failures, and at 20% variation the accuracy decreased to 65.625%. In contrast, the 8 × 128 configuration maintained 100% decryption accuracy even under 20% variation, demonstrating a sufficiently wide HD margin among encrypted outputs. The correlation map in Figure S9a shows a low average correlation coefficient of 0.139 among encrypted pairs, while the HD distribution for correctly decrypted pairs exhibits an average of 3.73%, compared to 18.75% for non‐matching LUT pairs (Figure S9b), thereby minimizing erroneous decryption. To statistically validate the hardware‐based cryptography, encryption and decryption were directly performed using the fabricated array through actual VMM operations. As shown in Figure 3f, the experimentally measured decryption accuracy improves markedly with increasing *N*, reaching 100% for the 8 × 128 configuration. Furthermore, the scheme was extended to MNIST handwritten digit encryption at the simulation level using a 784 × 12,544 crossbar array (Figure S10), demonstrating the feasibility of extending the proposed mapping to higher‐dimensional inputs. Importantly, this robustness is not solely a consequence of architectural scaling. The annealing‐engineered HRS landscape provides stabilized and diversified conductance distributions that effectively increase the separability of encrypted output vectors in the Hamming space. This materials‐level entropy directly enhances the HD margin among encrypted outputs, thereby suppressing output ambiguity and improving tolerance to conductance variations. Consequently, the reliability of the unified cryptographic framework is governed by the synergistic interplay between column‐dimensional scaling and engineered physical state distributions within the memristor array. While the simulation conceptually utilized a 784 × 12,544 array, this configuration was employed as a system‐level analysis to examine whether the proposed mapping and HD‐based recovery scheme maintains sufficient output separation when extended to higher‐dimensional input data, rather than to represent a directly fabricable monolithic array. In a practical hardware implementation, a matrix of this scale would need to be partitioned across multiple smaller crossbar tiles using spatial partitioning and/or time‐multiplexed operation. Furthermore, direct extension of the demonstrated scheme to large input widths using a single LUT would be impractical because the LUT size scales exponentially with the input width. The proposed architecture can instead employ fixed‐width block processing, in which a larger payload is partitioned into smaller input blocks that are processed sequentially or mapped onto parallel subarrays. For example, a 128‐bit payload can be divided into sixteen 8‐bit blocks, for which the LUT associated with each block is bounded to 2^8^ = 256 entries rather than requiring a monolithic 2^128^‐entry LUT. Combined with tiled crossbar implementation, this block‐wise approach provides a practical architectural pathway for extending the demonstrated 8‐bit scheme to larger input dimensions without requiring a single exponentially large LUT or monolithic crossbar.

### 2.3 Unified Entropy Generation: PUF and TRNG Performance

The array's security performance in PUF mode was first examined to verify its capability to generate stable, unique, and statistically unbiased hardware identities. As shown in Figure 4a, the responses from 10 independently configured array instances exhibit a near‐ideal balance between logic 0 and 1, yielding an average uniformity of 49.89% (σ = 0.15%) under 3‐bit ADC digitization. Such balanced response statistics are essential for maximizing entropy in weak PUF architectures and preventing bias‐induced predictability. The BL current distributions across all applied challenges (Figure S11a) are effectively translated into random‐looking binary outputs (Figure S11b), indicating that the annealing‐engineered conductance dispersion is effectively translated into statistically unbiased digital outputs. The uniqueness and reliability of the generated responses were further quantified through inter‐ and intra‐HD analyses, as shown in Figure 4b. An average inter‐HD of 49.91% approaches the theoretical ideal of 50%, confirming strong distinguishability among different PUF instances. Meanwhile, repeated measurements over 100 cycles yielded an average intra‐HD of 0.79%, corresponding to a bit error rate (BER) below 1%, thereby demonstrating excellent reproducibility of the hardware identity. The array resistance distribution maps of the 10 PUF instances (Figure S12) further reveal distinct spatial conductance landscapes across different array realizations, providing a physical basis for the observed inter‐instance uniqueness. The autocorrelation function (ACF) analysis in Figure 4c further verifies the absence of temporal dependency in the 10k‐bit response stream, with all ACF values remaining within the 95% confidence bounds (±0.0196). Collectively, these results demonstrate that the stabilized HRS landscape enables statistically independent, highly unique, and temporally stable PUF responses at the array level.

**FIGURE 4:**
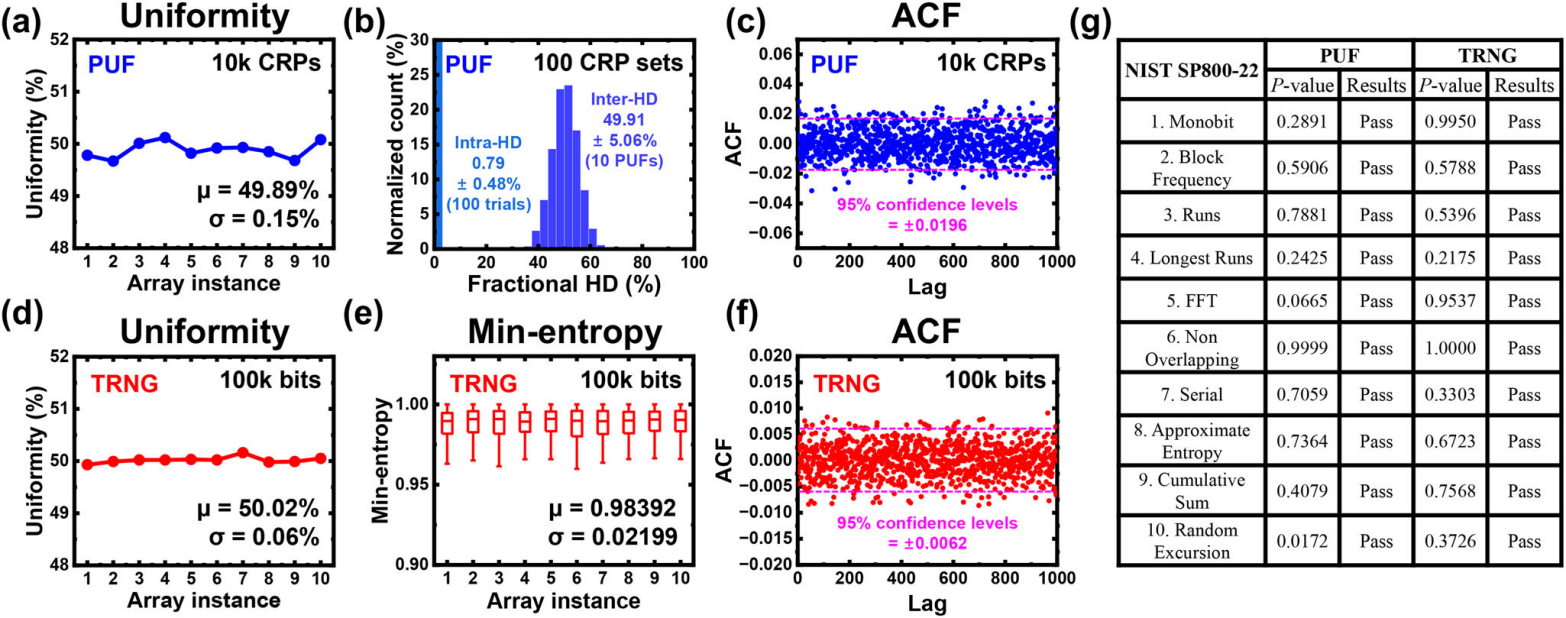
Unified static and dynamic entropy validation. (a) Uniformity of 1‐bit PUF responses across 10 array instances, demonstrating near‐ideal balance required for unbiased hardware identity generation. (b) Inter‐ and intra‐HD distributions for 128‐bit PUF responses, confirming high inter‐instance uniqueness and low intra‐instance variability. (c) ACF analysis of the PUF bitstream, verifying statistical independence and absence of temporal correlation. (d) Uniformity of a 100k‐bit TRNG sequence generated from RTN fluctuations, indicating negligible bias in dynamic entropy extraction. (e) Extracted minimum entropy approaching the theoretical limit, validating high‐quality random number generation. (f) ACF evaluation of TRNG output confirming negligible correlation among successive bits. (g) NIST SP800‐22 statistical test results for both PUF and TRNG modes, demonstrating compliance with standard randomness criteria.

In TRNG mode, the array operates as a dynamic entropy generator by exploiting RTN fluctuations originating from electron capture and emission events within individual memristor cells. As shown in Figure 4d, a 100k‐bit sequence generated from 10 array instances exhibits a uniformity of 50.02%, indicating negligible bias in the produced random stream. The measured RTN signals (Figure S13a) display clear two‐level stochastic transitions, which are processed through the BI TRNG circuit to generate output bits at transition edges (Figure S13b). This edge‐based digitization effectively converts analog noise into statistically robust digital randomness. The extracted bitstream achieves an average minimum entropy of 0.98392 (Figure 4e), approaching the theoretical ideal value of 1, thereby confirming the high‐entropy quality of the dynamic noise source. Temporal independence of the TRNG output was further validated through autocorrelation analysis of the 100k‐bit sequence (Figure 4f), where all values remain within the 95% confidence bounds (±0.0062), indicating negligible correlation among successive bits. To comprehensively evaluate randomness quality, both PUF responses and TRNG bitstreams were subjected to the NIST SP800‐22 statistical test suite. As summarized in Figure 4g, all 10 subtests were successfully passed in both operational modes, with p‐values satisfying the required criteria. These results collectively confirm that the annealed memristor crossbar array provides both static entropy for identity generation and dynamic entropy for high‐quality random number production within a unified hardware platform.

To further elucidate the physical origin of the dynamic entropy, temperature‐dependent RTN measurements were conducted to characterize the underlying trap dynamics (Figure S14). The mean electron capture and emission times were analyzed using the Arrhenius relationship. Linear fitting of the characteristic times as a function of reciprocal temperature yielded activation energies of approximately 0.32 and 0.37 eV for the capture and emission processes, respectively. These values are consistent with localized vacancy‐related trap states in the Al_2_O_3_ switching layer, supporting the interpretation that the temporal stochasticity utilized for TRNG generation originates from thermally activated electron trapping and detrapping. These results further indicate that the defect landscape stabilized by post‐fabrication annealing can preserve microscopic trap dynamics required for RTN‐based dynamic entropy generation. Notably, both the static entropy (PUF) and dynamic entropy (TRNG) originate from the same annealing‐engineered conductance landscape, highlighting the unified physical origin of the security primitives. Beyond its role as a dynamic entropy source, the TRNG also introduces session‐dependent randomness into the cryptographic operation. To evaluate the sensitivity of the encryption output to the TRNG‐generated nonce, identical plaintexts were repeatedly encrypted using different nonces. As shown in Figure S15, different nonces applied to the same plaintext produce distinct ciphertexts with large pairwise HDs. This nonce‐dependent behavior prevents deterministic reuse of an identical plaintext–ciphertext mapping and increases the difficulty of constructing a reusable static mapping from previously observed pairs. These results demonstrate that the dynamic entropy generated by the TRNG provides an additional source of temporal variability for the proposed physical security primitive.

To ensure the practical viability of the proposed hardware security platform, the reliability and temporal stability of the underlying device and entropy characteristics were further considered. At the device level, the 250°C‐annealed memristors maintain distinguishable HRS and LRS over 5 × 10^4^ switching cycles and exhibit stable resistance‐state retention for 10^4^ s at 100°C (Figure S8c,d), providing a reliable physical basis for repeated security operations. For the static entropy utilized in PUF operation, repeated response measurements yield a low average intra‐HD of 0.79% over 100 measurement cycles, demonstrating high reproducibility under repeated readout. For the dynamic entropy utilized in TRNG operation, the RTN originates from microscopic electron trapping and detrapping at localized vacancy‐related states rather than macroscopic filament reconfiguration. The resulting bitstreams exhibit near‐ideal uniformity, high minimum entropy, and negligible temporal correlation across the evaluated array instances, supporting robust dynamic entropy generation under the investigated operating conditions. Collectively, these device‐ and function‐level evaluations demonstrate that the annealed memristor array provides a reliable physical platform for both static and dynamic entropy‐based security operations.

### 2.4 Hardware‐Embedded Privacy‐Preserving Mutual Authentication (PPMA)

Building upon the previously demonstrated encryption/decryption architecture, we integrated the PUF and TRNG functionalities to develop a lightweight mutual‐authentication framework, termed PPMA, as illustrated in Figure 5a. Since data exchange between the server and the IoT mote occurs over an unsecured communication channel, directly transmitting plaintext would introduce significant security vulnerabilities. In the proposed protocol, the server‐side TRNG first generates two random streams, C_1_ and C_2_. These values are processed through the unified crossbar array, where C_1_ is applied to the PUF to generate response R_1_, and C_2_ is encrypted to produce Enc(C_2_). The server then transmits Enc(C_2_) together with C_1_ ⊕ C_2_, thereby preventing exposure of raw plaintext over the insecure channel.

**FIGURE 5:**
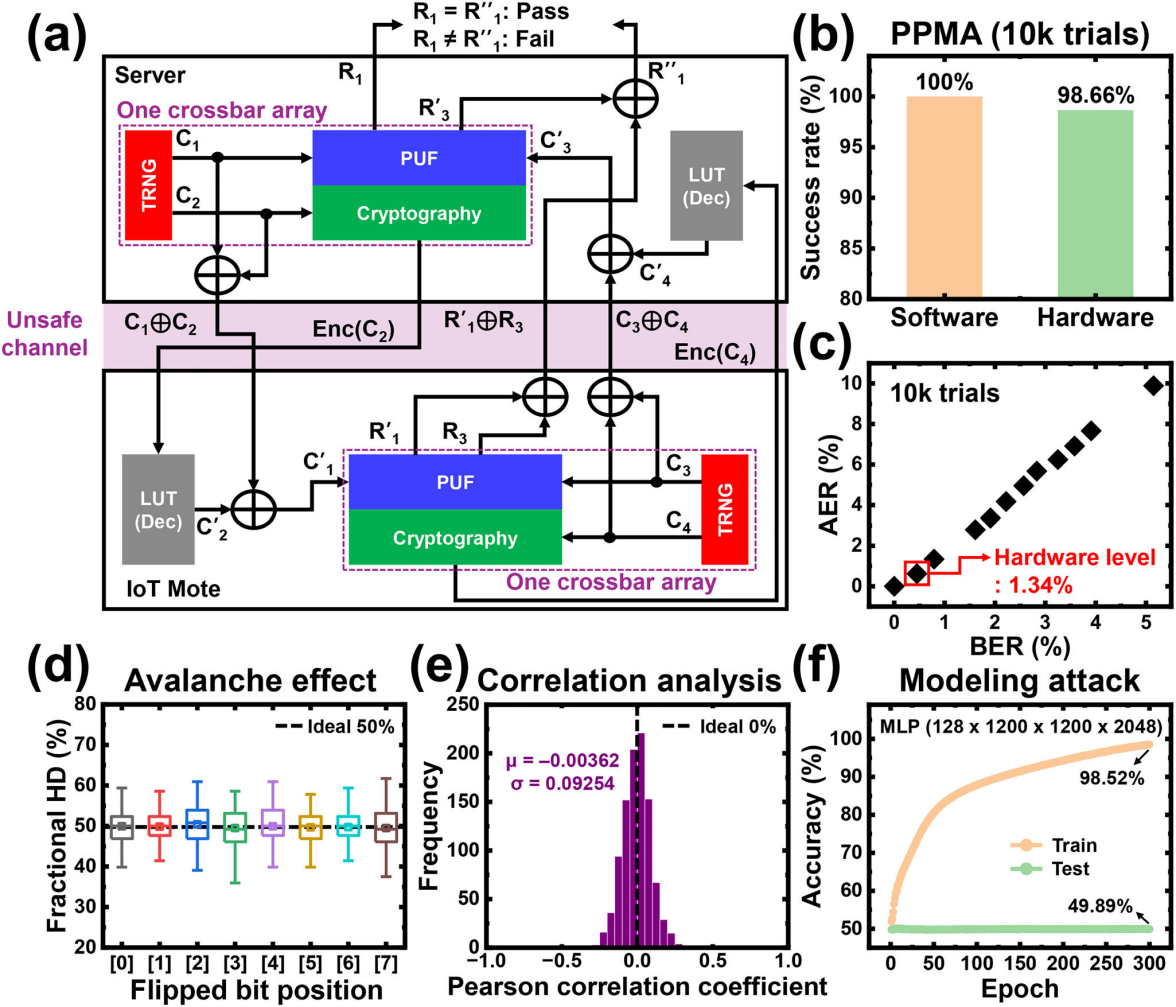
Hardware‐embedded PPMA and security analysis assessment. (a) Schematic of the PPMA protocol integrating PUF, TRNG, and in‐memory cryptographic processing within a single crossbar array, preventing direct exposure of plaintext over insecure channels. (b) Mutual authentication success rate over 10,000 trials under ideal and hardware‐emulated conditions, demonstrating strong practical reliability. (c) AER vs. PUF BER, showing controlled error amplification and robust authentication performance in the optimized 8 × 128 configuration. (d) Avalanche effect evaluation, achieving an ideal fractional HD of ∼50% against 1‐bit input flips. (e) Correlation analysis of ciphertexts, exhibiting a near‐zero mean Pearson correlation coefficient, indicating negligible linear correlation among the evaluated ciphertexts. (f) Evaluation against an MLP‐based modeling attack, showing near‐random‐guess prediction accuracy (∼50%) for unseen test samples under the evaluated attack configuration.

Upon reception, the IoT mote decrypts Enc(C_2_) to recover C_2_′ using the LUT and HD‐based matching and subsequently derives C_1_′ from the received XORed data. The recovered C_1_′ is applied to the mote's PUF to generate R_1_′. In parallel, the mote TRNG produces a new pair of random streams, C_3_ and C_4_, which are processed to generate response R_3_ and encrypted output Enc(C_4_). The mote transmits Enc(C_4_) along with C_3_ ⊕ C_4_ and R_3_ ⊕ R_1_′ back to the server. Finally, the server reconstructs C_3_′ and R_1_″ through decryption and XOR operations, and mutual authentication is completed by comparing R_1_″ with the originally generated R_1_. The core PUF, TRNG, and analog in‐memory encryption primitives are co‐realized within the same memristor crossbar array, with LUT/HD‐assisted decryption and protocol control performed externally. This architecture enables compact and hardware‐efficient mutual authentication while reducing reliance on externally stored digital secret keys and dedicated security accelerators.

To evaluate the practical applicability of the proposed PPMA framework, mutual authentication experiments were conducted by incorporating the integrated PUF, TRNG, and encryption/decryption mechanisms into the full protocol. As shown in Figure 5b, 10,000 authentication trials under ideal conditions yielded a 100% success rate, while hardware‐based emulation achieved a success rate of 98.66%, indicating strong resilience against device‐level variations and circuit non‐idealities. It should be noted that the 98.66% authentication success rate represents the raw performance obtained from the hardware‐emulated responses without applying error correction or repeated‐response post‐processing. This value therefore reflects the intrinsic reproducibility of the physical hardware rather than a post‐processed system‐level authentication rate. In practical implementations, established response‐stabilization techniques, such as error‐correcting codes or repeated‐read majority voting, could be incorporated to further improve authentication reliability. Figure 5c further presents the authentication error rate (AER) as a function of the PUF BER. Because the PUF and cryptographic operations are realized within the same array, the intrinsic BER influences multiple stages of the authentication procedure, including two PUF evaluations and two encryption/decryption processes. This accumulation results in an amplified AER. Nevertheless, in the optimized 8 × 128 configuration—where 100% decryption accuracy was previously confirmed despite repeated operations (Figure S16)—the AER scales to approximately twice the intrinsic BER, indicating that encryption/decryption reliability remains well preserved. Under hardware‐measured conditions, a BER of 0.79% corresponds to an AER of only 1.34%. These results demonstrate that the proposed PPMA protocol maintains robust authentication performance even under realistic hardware variations, highlighting its suitability for compact, low‐power IoT security systems.

To quantitatively characterize the security‐related properties of the proposed physical primitive, we first evaluated its input sensitivity and ciphertext decorrelation. The avalanche effect was examined by monitoring the output ciphertext variation when a single bit of the input was flipped. As shown in Figure 5d, a 1‐bit change in the input yields a fractional HD approaching the ideal value of 50%, demonstrating strong sensitivity of the ciphertext to small changes in the input. Furthermore, we evaluated the correlation between ciphertexts generated from adjacent or highly similar inputs. As illustrated in Figure 5e, the Pearson correlation coefficient distribution is tightly centered near zero, indicating negligible linear correlation among the evaluated ciphertexts. Together, these results demonstrate pronounced avalanche behavior and low linear correlation among the evaluated ciphertexts. We further evaluated the resistance of the proposed physical mapping to machine‐learning‐assisted modeling attacks. A multilayer perceptron (MLP) network was trained to predict the ciphertexts based on collected input–output pairs. For this analysis, a 128 × 2,048 VMM‐based encryption architecture conceptually composed of sixteen 8 × 128 array blocks was considered. As presented in Figure 5f, the model achieves a training accuracy of approximately 98% with 1,000 training samples, whereas the prediction accuracy for 100 previously unseen test samples remains near the random‐guess level of 50%. The substantial difference between the training and test accuracies indicates that the evaluated MLP predominantly learns the training samples but exhibits limited generalization to unseen input–output relationships under the present experimental conditions.

Moreover, the resilience of the proposed PPMA protocol against replay and payload‐manipulation scenarios was evaluated using the protocol‐level simulation shown in Figure S17. In the normal authentication session, a legitimate request generated using a fresh TRNG nonce is successfully authenticated. In the exact replay scenario, previously intercepted authentication data are reused; although the reconstructed response itself retains an HD of 0%, the replayed request is rejected through verification of the session‐dependent nonce information. In the payload‐manipulation scenario, the intercepted ciphertext is combined with a different nonce, resulting in a mismatch between the reconstructed and expected responses and increasing the HD to 51.6%, thereby causing authentication failure. These results demonstrate the ability of the proposed PPMA protocol to distinguish legitimate authentication from the evaluated replay and payload‐manipulation scenarios. In addition, the proposed analog in‐memory encryption minimizes data transfer between the memory array and external processing units by performing the VMM‐based cryptographic transformation directly within the crossbar. This reduced data movement may provide an architectural advantage in limiting some conventional bus‐related information leakage; however, a dedicated power‐ or electromagnetic‐side‐channel analysis would be required to quantitatively assess such resistance. Finally, unlike conventional cryptographic schemes that explicitly store a digital key, the proposed physical mapping is defined by the device‐specific conductance configuration of the memristor array, while TRNG‐generated nonces provide session‐dependent randomness. This architecture reduces reliance on externally stored static digital keys and may therefore mitigate vulnerabilities associated with conventional digital key storage.

To clarify the architectural distinction of the proposed platform, Table S1 compares this work with state‐of‐the‐art memristor‐based hardware security implementations. Several recent works have demonstrated integrated PUF and TRNG functionalities using 1T1R, passive, or 3D memristive architectures. Other studies have implemented memory‐based cryptographic operations, including XOR‐ and hash‐based approaches. Building upon these complementary advances, the present work combines static and dynamic entropy generation with analog VMM‐based in‐memory encryption within a common passive crossbar platform. This integration enables the implementation of the PPMA protocol while reducing the need to transfer intermediate security data between functionally separated hardware modules.

## 3 Conclusion

In summary, we have demonstrated a unified hardware security platform that co‐realizes PUF, TRNG, and analog in‐memory encryption within a single annealed memristor crossbar array and integrates these primitives with LUT/HD‐assisted decryption for PPMA. By engineering the HRS landscape through controlled post‐fabrication annealing, a stabilized dual‐entropy material state was established, simultaneously supporting static device‐to‐device variability for PUF operation and dynamic RTN for TRNG functionality. This materials‐level entropy provides the physical foundation for secure and statistically robust hardware security primitives. Through architectural scaling of the column dimension in the 8 × *N* configuration, collision probability was effectively suppressed by expanding the effective physical bit space of the crossbar array, enabling reliable and variation‐tolerant in‐memory encryption and decryption. Comprehensive statistical evaluations, including HD analysis, autocorrelation testing, and NIST SP800‐22 assessments, confirmed high‐quality entropy generation in both static and dynamic modes. Building upon this unified entropy platform, the proposed PPMA protocol achieved robust mutual authentication performance, maintaining high reliability even under realistic device‐level variations. The proposed framework establishes a materials‐engineered pathway toward compact, low‐power, and highly integrated hardware security systems. By consolidating entropy generation, cryptographic processing, and authentication within a single physical array, this approach reduces architectural complexity while enhancing intrinsic security. These results highlight the potential of annealed memristor crossbar arrays as scalable and practical hardware security primitives for next‐generation IoT applications. Beyond memristor crossbars, the proposed materials‐to‐security integration strategy may be extended to other emerging memory platforms where engineered disorder and intrinsic variability can be harnessed as functional security resources.

## 4 Experimental Methods

### 4.1 Memristor Crossbar Fabrication Optimization

The fabrication of the memristor crossbar array started with the thermal oxidation of a 4‐inch silicon wafer to form a 300 nm‐thick SiO_2_ insulating layer, which served as the substrate. BE patterns were defined using photolithography with an aligner, employing approximately 2 µm‐thick negative photoresist engineered with an undercut profile to facilitate a reliable lift‐off process. The spacing between adjacent electrode lines was maintained at 5 µm to ensure sufficient pattern separation. To form the BE stack, a bilayer consisting of a 10 nm Ti adhesion layer and a 50 nm Pt layer was deposited by e‐beam evaporation, followed by lift‐off in acetone to define the electrode geometry. An ultrathin Al_2_O_3_ film, serving as the resistive switching layer, was subsequently deposited by atomic layer deposition. The deposition was carried out at 450°C for 18 cycles using trimethylaluminum (TMA) as the Al precursor and ozone (O_3_) as the oxygen reactant, resulting in a film thickness of approximately 1.53 nm. Following the formation of the switching layer, the TiO_x_/TiO_y_ film was deposited as an oxygen reservoir and rich layers, respectively, by in situ direct current reactive sputtering using a Ti target in an Ar/O_2_ mixed atmosphere at room temperature and a chamber pressure of 10 mtorr, where the Ar flow rate was set to 30 sccm. The O_2_ flow rates during the deposition of the TiO_x_ and TiO_y_ layers were set to 0.7 and 1.0 sccm, respectively. Under these conditions, the TiO_x_ layer was deposited for 300 s, resulting in a thickness of 17.2 nm, while the TiO_y_ layer was deposited for 60 s to achieve a thickness of 4.0 nm, corresponding to deposition rates of 0.573 and 0.667 A/s, respectively. After that, TEs were fabricated using the same photolithography and lift‐off process as the BEs. A 5 nm Ti layer was first deposited as an adhesion layer, followed by an 80 nm Pt layer via e‐beam evaporation. To define the active switching region, the switching layer was selectively etched using CHF_3_ plasma for 30 s to expose the underlying BEs. Consequently, each memristive device was formed at the cross‐point between the BE and TE lines, resulting in an active device area of 2.5 × 2.5 µm^2^. Finally, a post‐annealing process was carried out at temperatures ranging from 200°C to 350°C for 10 min under N_2_ ambient to thermally activate interfacial scavenging, thereby enabling forming‐free switching characteristics. The completed memristor crossbar arrays were finally evaluated through comprehensive electrical and structural characterizations to verify device performance and integrity.

### 4.2 Electrical Characterizations

The electrical characteristics of the fabricated memristor crossbar array were characterized using a Keysight B1500A semiconductor parameter analyzer equipped with a source/measure unit (SMU) and a waveform generator/fast measurement unit (WGFMU), connected with a Keysight 34980A switching matrix. To establish physical contact with the 64 electrodes, a custom‐designed 64‐pin probe card was connected to the extended electrode pads of the fabricated memristor crossbar array, enabling the application of electrical signals to each electrode. Array connection control and voltage signal application were managed by a PC through custom C++‐based commands. During the write operation, voltage pulses were applied using the WGFMU under a half‐V biasing scheme, whereas during the read operation, DC voltages were supplied by the SMU under a ground scheme. Exceptionally, for tracking RTN signals in the memristor devices, current transient curves were extracted and analyzed using *V*_read_ pulses generated by the WGFMU.

### 4.3 Encryption/Decryption with Unified PUF and TRNG

To demonstrate a lightweight security system, the encryption and decryption functionality implemented using a hardware memristor crossbar array with the unified PUF and TRNG capabilities was experimentally verified through electrical measurements. In the reconfigured 8 × *N* array, 8‐bit binary random values were applied to the WLs as input, where 0 and 1 were assigned to ground and *V*_read_, respectively. To realize a larger effective *N* along the column direction within the limited 32 × 32 size of the fabricated crossbar array, the same input sequence was repeatedly applied. For example, when the array size was 8 × 32 or smaller, the input sequence was applied once, whereas for reconfigured arrays corresponding to larger values of *N*, the same input sequence was applied two or more times. As a result, the full utilization of all 1,024 memristor cells in the fabricated crossbar array was achieved when *N* reached 128. After applying an 8‐bit multi‐input sequence for the PUF and encryption operations, the resulting BL current was converted into a voltage using an inverting‐amplifier‐based TIA, where the resistance of 100 kΩ was selected for *R*_i1_ to appropriately translate bit‐line currents in the microampere range into voltages within the supply voltage (*V*_DD_ = 5 V), whereas *R*_i2_ was set to 1 MΩ for TRNG. After that, to shift the determined negative output voltage (*V*_out_) to a positive voltage suitable for ADC input, a resistive voltage divider was employed, where *R*_o1_ and *R*_o2_ were assigned to 300 and 100 kΩ, respectively. The ADC resolution window was assumed to be adaptively determined according to the HRS distribution of the designed memristor crossbar array. In addition, for the TRNG circuit used in BI random number generation, the passive components *C*_i1_, *C*_o1_, and *R*_o3_ were selected as 220 µF, 30 µF, and 10 kΩ, respectively, based on previous work, and all other parameters were assumed to be the same as those of the existing BI TRNG circuit [66]. For TRNG operation, individual memristor cells were randomly selected and accessed, and their RTN characteristics were extracted through ground‐scheme‐based read operations with a *V*_read_ pulse. The extracted RTN signals were then processed by a BI TRNG circuit to generate random numbers. Finally, the decryption operation was implemented using a LUT, in which the input corresponding to the output with the minimum HD was returned. If two or more outputs exhibited the same minimum HD, the result was regarded as an incorrect decryption. The decryption process can be readily implemented using simple XOR gate operations and a counter; in this work, it was carried out in software.

### 4.4 Lightweight PPMA Protocol Environments

Based on the integrated hardware security array, a lightweight PPMA was designed, and hardware parameters were applied to evaluate the practical lightweight mutual authentication performance and reliability trends. In this system, the CRPs and the LUT corresponding to the integrated security crossbar array were assumed to be pre‐stored at the server. The increasing trend of the AER with respect to the BER of the PUF was verified through simulations based on 10,000 authentication attempts; here, the XOR operation used to prevent CRP exposure was assumed to be ideal. In addition, the hardware error level was estimated from the variations (up to 0.6%) in the extracted weights observed during repeated read operations. In the bidirectional mutual authentication scheme, the PUF BER contributed to approximately a twofold increase in the AER, and the feasibility of the lightweight authentication system was demonstrated.

## Conflicts of Interest

The authors declare no conflicts of interest.

## Supporting information




**Supporting File**: smll75550‐sup‐0001‐SuppMat.docx.

## Data Availability

The data that support the findings of this study are available from the corresponding author upon reasonable request.
