# Supplementary material for: Unified In‐Memory Hardware Security Enabled by an Entropy Landscape in a Memristor Crossbar
Source: Small. 2026 Sep 2;22(62):e75550. doi: 10.1002/smll.75550 (PMC13742895; doi:10.1002/smll.75550)
Supplement: Supplementary file 1 [file SMLL-22-e75550-s001.docx]

Supporting Information

**Unified In-Memory Hardware Security Enabled by an Entropy Landscape in a Memristor Crossbar**

Jinwoo Park and Hyungjin Kim

Division of Materials Science and Engineering and Department of Semiconductor Engineering, Hanyang University, Seoul 04763, South Korea

Corresponding author: Hyungjin Kim (hkim12@hanyang.ac.kr).

**Figure S1.** **First reset operation of thermally formed cells in the 32 × 32 memristor crossbar array under ISPP.** (a–d) Representative first-reset characteristics after annealing at 200, 250, 300, and 350 °C, respectively. With increasing annealing temperature, a progressively larger fraction of devices initiates switching from the first reset operation without requiring an initial forming set transition, reflecting enhanced thermal activation and vacancy generation within the switching layer. The number of thermally formed cells increases from 15 at 200 °C to 443 at 350 °C, indicating a transition toward a forming-free switching regime at elevated temperatures. To consistently identify thermally activated cells under varying pristine conductance levels, the reset threshold current was set to 1, 3, 5, and 7 μA for 200, 250, 300, and 350 °C, respectively.

**Figure S2. Resistance colormaps of the (a) pristine state and (b) HRS after switching cycles under different annealing temperatures.** With increasing annealing temperature, thermal scavenging at the interfaces promotes oxygen vacancy generation, progressively lowering the pristine resistance and facilitating thermal activation of the switching layer. These vacancies reduce the forming voltage and mitigate stochastic overshoot-induced hard breakdown observed at lower annealing temperatures. The HRS maps were obtained after 10 consecutive set–reset cycles starting from the pristine state using the ISPP voltage sequence shown in the inset of Figure 2g. While insufficient annealing results in broader resistance dispersion and occasional abnormal cells due to incomplete activation, excessive annealing can induce aggressive interfacial reactions that destabilize reset behavior. The 250 °C condition yields the most uniform resistance landscape, supporting stable array-level switching and forming the materials foundation for reliable entropy generation.

**Figure S3. Array-level switching robustness evaluated under different annealing temperatures.** The switching capability of all 1,024 memristor cells in the 32 × 32 array was assessed by comparing the initial resistance distribution with that obtained after 10 consecutive set–reset cycles using the ISPP scheme. Cells that failed to switch or remained permanently in the low-resistance state were classified as failed devices. Across all annealing conditions, high array yields exceeding 99% were achieved, indicating effective suppression of catastrophic switching failures through post-fabrication annealing. Notably, the 250 °C condition exhibited the highest yield with no observed failed cells, reflecting an optimal balance between sufficient thermal activation and controlled interfacial reactions. Both lower and higher annealing temperatures showed a small fraction of failures, consistent with competing mechanisms of incomplete activation and excessive thermal forming discussed in the main text.

**Figure S4. Evolution of read current during ISPP-based reset operation for representative reset-failure cases under different annealing conditions: (a) as-deposited, (b) 200 °C, (c) 300 °C, and (d) 350 °C.** The 250 °C condition is excluded as no reset failure was observed under this optimized annealing temperature. In the as-deposited state, stochastic overshoot currents during forming induce excessively strong filament formation within the switching layer, resulting in an abnormally low resistance state. Consequently, the applied reset voltage cannot be effectively redistributed across the switching region, leading to irreversible reset failure. A similar failure mode is observed at 200 °C, where insufficient thermal activation and weak interfacial reactions between TiO_x_ and TiO_y_ promote probabilistic overshoot events and the formation of unintended auxiliary conductive paths. These unstable conduction channels cause pronounced current fluctuations during reset, preventing proper resistance recovery. In contrast, at higher annealing temperatures (≥300 °C), excessive thermal activation and enlarged interfacial overlap at the TiO_x_/TiO_y_ interface lead to aggressive thermal forming. The competition between the main switching layer and the interfacial conduction region produces abrupt current surges in the opposite polarity during reset, destabilizing the reset process. These distinct failure signatures highlight the competing mechanisms associated with insufficient and excessive annealing, reinforcing the existence of an optimal annealing window around 250 °C for stable array-level switching.

**Figure S5. Annealing-temperature-dependent *I*–*V* characteristics of memristor cells: (a) as-deposited, (b) 200 °C, (c) 300 °C, and (d) 350 °C.** The optimized 250 °C condition is presented in Figure 2f of the main text. In the as-deposited state, the Al_2_O_3_ switching layer remains relatively trap-deficient and structurally intact, resulting in a pronounced voltage gap between the initial forming and subsequent set processes. As the annealing temperature increases, thermally induced interfacial scavenging between the switching layer and the oxygen reservoir layer generates additional oxygen vacancies, effectively introducing trap states and reducing the forming–set voltage disparity. This progressive thermal activation promotes forming-free switching behavior. However, at excessively high annealing temperatures (≥300 °C), over-generation of vacancy-related trap states leads to aggressive thermal forming, where devices initiate switching from the first reset operation. The resulting reduction in switching-layer resistance lowers the overall cell resistance and destabilizes reset dynamics, increasing the likelihood of reset failure. These results further confirm the existence of an optimal annealing window, around 250 °C, that balances sufficient thermal activation with controlled interfacial reactions to achieve stable and reliable switching.

**Figure S6. XPS analysis of the constituent titanium oxide layers in the as-deposited state.** Ti 2p and O 1s core-level spectra of the (a) TiO_x_ and (b) TiO_y_ layers. Both layers exhibit characteristic Ti^4+^-related Ti 2p peaks at approximately 458 eV and O 1s peaks at approximately 530 eV. Quantitative analysis of the XPS spectra yields O/Ti atomic ratios corresponding to approximately TiO_1.67_ and TiO_1.75_ for the TiO_x_ and TiO_y_ layers, respectively. The lower O/Ti ratio of the TiO_x_ layer indicates a greater degree of oxygen deficiency relative to the TiO_y_ layer, supporting a higher relative abundance of oxygen-vacancy-related defects.

**Figure S7. Conduction mechanism analysis of the memristive devices under different annealing conditions.** (a) ln(*I*) versus *V*^1/2^ plots for the HRS. The linear dependence is consistent with Schottky emission, indicating interface-limited charge transport in the HRS. (b) ln(*I*) versus ln(*V*) plots for the LRS. The linear dependence with a slope of approximately 1 indicates Ohmic conduction, consistent with transport through a filamentary conductive pathway. Similar conduction behaviors are observed across the investigated annealing conditions, supporting a common filamentary switching mechanism despite the annealing-dependent changes in device characteristics.

**Figure S8. Supplementary switching stability and reliability characteristics of the 250 °C-annealed memristor crossbar array.** (a) Representative *I*–*V* characteristic of a thermally formed cell observed at 250 °C due to local annealing-temperature variation. Cells exhibiting relatively high initial current levels probabilistically initiated switching from the first reset operation, reflecting partial thermal activation within the array. (b) Distribution of HRS and LRS currents for all 1,024 cells in the 32 × 32 array. The average HRS and LRS currents were 2.36 μA (σ = 1.01 μA) and 12.9 μA (σ = 3.63 μA), respectively. While the HRS shows a broader relative variability (42.8%) compared to the LRS (28.1%), a clear resistance window is maintained across the array, enabling reliable readout and robust entropy extraction. (c) Endurance characteristics measured over 5 × 10^4^ switching cycles, demonstrating stable resistance modulation without progressive degradation. (d) Retention performance of both resistance states for 10^4^ s at 100 °C, confirming nonvolatile stability of the stored conductance states. These results indicate that the optimized 250 °C annealing condition ensures sufficient resistance contrast, statistical dispersion for entropy generation, and long-term switching reliability, collectively supporting stable operation of the unified hardware security framework.

**Figure S9. Statistical analysis of encrypted outputs and decryption margins in the 8 × 128 configuration.** (a) Colormap of Pearson correlation coefficients among encrypted bitstreams generated from different input patterns. Excluding the diagonal self-correlations, the average correlation coefficient is 0.139, indicating weak mutual dependence and minimal structural similarity between encrypted outputs. This low correlation reflects effective utilization of the engineered conductance dispersion in producing decorrelated ciphertexts. (b) HD distributions between correctly decrypted pairs and non-matching LUT pairs. Correctly decrypted pairs exhibit a tightly clustered low HD distribution (μ = 3.73%, σ = 2.27%), while non-matching pairs show a significantly higher HD distribution (μ = 18.75%, σ = 3.69%), establishing a clear separation margin. This wide HD gap minimizes the probability of erroneous LUT selection and underpins the variation tolerance of the proposed in-memory cryptographic scheme. Increasing the column dimension further expands this HD margin, thereby enhancing decryption robustness.

**Figure S10.** **System-level simulation of MNIST image encryption and decryption using a large-scale memristor crossbar array.** A binarized 28 × 28 MNIST handwritten digit (784-bit input) was encrypted using a simulated 784 × 12,544 crossbar configuration, where the column dimension was set to be 16 times larger than the row dimension to suppress collision probability in high-dimensional output space. The resulting encrypted data forms a decorrelated random binary matrix with no visually identifiable structure. Decryption was successfully performed using the LUT and HD similarity, fully reconstructing the original input image. These results demonstrate the scalability of the proposed in-memory cryptographic framework to large input dimensions and confirm that expanding the column space effectively preserves collision-free and reliable decryption behavior.

**Figure S11. Raw current distribution and corresponding binary response map in PUF mode.** (a) BL current distributions measured for all applied challenge inputs under PUF operation. The ADC resolution was selected according to the dynamic current range to ensure balanced and reliable binary quantization, thereby minimizing bias in the digitized responses. (b) Binary response map constructed from 1-bit outputs corresponding to all challenge inputs. The implemented weak-PUF architecture supports 2^8^ × 128 = 32,768 CRPs, leveraging the crossbar’s column scaling to expand the available identity space. The resulting response map exhibits no discernible spatial pattern, reflecting statistically unbiased entropy generation consistent with the uniformity and HD results presented in Figure 4.

**Figure S12.** **Array-level resistance colormaps corresponding to 10 independent PUF instances fabricated using 32 × 32 memristor crossbar arrays annealed at 250 °C.** Each instance exhibits a distinct HRS distribution arising from intrinsic device-to-device variability within the engineered resistance landscape. Although the global resistance statistics remain similar across arrays, the spatial resistance patterns are decorrelated, generating unique PUF response fingerprints for each instance. This instance-level resistance diversity provides the physical origin of the near-ideal inter-HD performance reported in Figure 4b of the main text.

**Figure S13. RTN characteristics and corresponding TRNG outputs from representative memristor cells.** (a) RTN waveforms measured over 2 s from six randomly selected memristor cells within a BL of the crossbar array. The discrete current fluctuations originate from stochastic electron capture and emission events at vacancy-related trap sites, serving as an intrinsic dynamic entropy source for true random number generation. (b) Binary sequences (1 Mb) generated from the corresponding RTN signals of the six cells. The extracted TRNG outputs exhibit statistically balanced distributions of 0s and 1s with no discernible deterministic pattern, confirming that the RTN-driven entropy is effectively translated into unbiased digital randomness.

**Figure S14. Temperature-dependent extraction of activation energies associated with RTN in the 250 °C-annealed memristor.** Arrhenius plots of the mean (a) electron capture time and (b) electron emission time as a function of reciprocal temperature (1000/*T*). Linear fitting yields activation energies (*E*_a_) of approximately 0.32 and 0.37 eV for the capture and emission processes, respectively. These activation energies are consistent with localized vacancy-related trap states in the Al_2_O_3_ switching layer, supporting the interpretation that the observed RTN originates from thermally activated electron trapping and detrapping processes. The results further indicate that microscopic trap dynamics are preserved after post-fabrication annealing, providing the dynamic entropy utilized for TRNG operation.

**Figure S15. Evaluation of TRNG nonce sensitivity in the proposed encryption scheme.** Fractional HDs between ciphertexts generated from an identical plaintext using different TRNG nonces over 100 consecutive encryption instances. Despite the identical plaintext, dynamically changing the nonce produces distinct ciphertexts with fractional HDs distributed around 50%, demonstrating strong sensitivity of the encryption output to the TRNG-generated nonce. This nonce-dependent behavior prevents deterministic reuse of an identical plaintext–ciphertext mapping across encryption instances and increases the difficulty of constructing a reusable static mapping from previously observed pairs.

**Figure S16. Decryption reliability under repeated encryption/decryption evaluations.** The decryption error rate was evaluated by performing 100 consecutive read-based VMM encryption/decryption operations for all input patterns in the 8 × 128 configuration. Despite repeated analog VMM executions and accumulated read-induced variations, the decryption error rate remained at 0%, confirming the stability of the HD-based LUT matching and the robustness of the proposed in-memory cryptographic framework against operational fluctuations.

**Figure S17. Protocol-level evaluation of replay and payload-manipulation scenarios in the proposed PPMA scheme.** The simulation illustrates three representative authentication scenarios. In Session 1 (normal authentication), a legitimate request generated using a fresh TRNG nonce is successfully authenticated with a 0% HD. In Session 2 (exact replay), an adversary reuses the intercepted authentication data from the previous session. Although the reconstructed response retains an HD of 0%, the replayed request is rejected through the freshness verification of the previously used nonce. In Session 3 (modified nonce), the intercepted ciphertext is combined with a different nonce, resulting in a mismatch between the reconstructed and expected responses and increasing the HD to 51.6%, thereby causing authentication failure. These results illustrate how session-dependent nonce information enables the proposed PPMA protocol to distinguish legitimate authentication from the evaluated replay and payload-manipulation scenarios.

**Table S1. Comparison of state-of-the-art memristor-based hardware security primitives.**

|  | Yang et al.  [52] | Ding et al.  [49] | Gao et al.  [47] | Woo et al.  [53] | Kim et al.  [54] | Li et al.  [46] | This work |
| --- | --- | --- | --- | --- | --- | --- | --- |
| Device architecture  (Technology) | 1T1R  (40 nm) | Passive memristor | 1T1R  (40 nm) | Passive memristor | Passive memristor | 1T1R  (28 nm) | Passive memristor |
| Demo complexity | – | 4 × 8 × 32 | 4-kb | 4 × 4 | 9 × 9 | 32 × 32 | 32 × 32 |
| Unified entropy generation  (PUF + TRNG) | No | Yes | Yes | No | No | Yes | Yes |
| In-memory cryptographic operation | No | No | No | No | No | No | Yes |
| PUF entropy source | SS of transistor | Leakage mismatch | D2D variation between 2R | – | – | Forming voltage variation | HRS distribution |
| TRNG entropy source | – | Oscillation period Fluctuation | Read noise | – | – | Read noise | RTN |
| Encryption function | XOR | – | – | XOR | XOR  (dual-polarity) | Hash function | VMM with ADC |
| Decryption function | XOR | – | – | XOR | XOR  (dual-polarity) | – | LUT HD similarity |
| Uniformity/  Uniqueness/  BER | 49.7%/  49.95%/  5.06% | –/  49.97%/  1.65% | 50.03%/  50.00%/  0% | –/  –/  – | –/  –/  – | 50.09%/  50.06%/  0% | 49.89%/  49.91%/  0.79% |
| Uniformity/  Min-entropy | –/  – | 47.2%  /0.9984 | 43.85%  /0.9991 | –/  – | –/  – | 49.99%/  0.9893 | 50.02%/  0.9839 |
| NIST test  (PUF/TRNG) | –/– | –/Pass | Pass/Pass | – | – |  | Pass/Pass |
| System-level protocol | – | – | PPMA | – | – | PPMA | PPMA |

Note: PUF, physical unclonable function; TRNG, true random number generator; D2D, device-to-device; HRS, high-resistance state; RTN, random telegraph noise; VMM, vector–matrix multiplication; ADC, analog-to-digital converter; LUT, lookup table; HD, Hamming distance; BER, bit error rate; and PPMA, PUF-based privacy-preserving mutual authentication. “Unified entropy generation” denotes the implementation of both PUF and TRNG functionalities using a common memristive hardware platform, whereas “in-memory cryptographic operation” indicates that the memory device or array directly participates in the cryptographic data transformation rather than serving solely as a storage element. “Demo complexity” represents the experimentally demonstrated array or hardware scale reported in each reference and is not intended as a direct comparison of effective memory capacity. For PUF evaluation, uniformity represents the balance between binary responses, uniqueness quantifies the inter-device or inter-instance response difference, and BER characterizes the reproducibility of responses under repeated evaluation. For TRNG evaluation, uniformity represents the balance between generated 0 and 1 bits, while min-entropy quantifies the unpredictability of the generated random sequence. The “system-level protocol” category indicates whether the corresponding work demonstrated integration of the hardware security primitives into a higher-level authentication protocol. “–” indicates that the corresponding functionality or metric was not reported or evaluated in the referenced work. Performance metrics are presented as reported in the corresponding references under their respective experimental conditions and should therefore be interpreted as representative benchmarks rather than strictly normalized head-to-head comparisons.
